# Alcohol consumption and colorectal carcinogenesis: an exploration of the gut microbial pathway as a potential mediator

**DOI:** 10.1007/s00394-026-03960-6

**Published:** 2026-04-21

**Authors:** Ane Sørlie Kværner, Einar Birkeland, Ekaterina Avershina, Edoardo Botteri, Cecilie Bucher-Johannessen, Markus Dines Knudsen, Anette Hjartåker, Christian M. Page, Johannes R. Hov, Mingyang Song, Kristin Ranheim Randel, Geir Hoff, Trine B. Rounge, Paula Berstad

**Affiliations:** 1 Department of colorectal cancer screening, Cancer Registry of Norway, National Institute of Public Health, Oslo, Norway 5313 Majorstuen, 0304; 2 Department of Pharmacy, University of Oslo, Oslo, Norway; 3 Department of Informatics, University of Oslo, Oslo, Norway; 4 Department of Tumor Biology, Institute of Cancer Research, Oslo University Hospital, Oslo, Norway; 5 Department of Research, Cancer Registry of Norway, Norwegian Institute of Public Health, Oslo, Norway; 6 Department of Nutrition, Institute of Basic Medical Sciences, University of Oslo, Oslo, Norway; 7 Department of Epidemiology, Harvard T.H. Chan School of Public Health, Boston, MA USA; 8 Centre for Fertility and Health, Norwegian Institute of Public Health, Oslo, Norway; 9 Department of Physical Health and Ageing, Division of Mental and Physical Health, Norwegian Institute of Public Health, Oslo, Norway; 10 Department of Transplantation Medicine, Division of Surgery and Specialized Medicine, Norwegian PSC Research Center, Oslo University Hospital, Oslo, Norway; 11 Research Institute of Internal Medicine, Division of Surgery and Specialized Medicine, Inflammatory Diseases and Transplantation, Oslo University Hospital, Oslo, Norway; 12 Faculty of Medicine, Institute of Clinical Medicine, University of Oslo, Oslo, Norway; 13 Section of Gastroenterology, Department of Transplantation Medicine, Division of Surgery and Specialized Medicine, Oslo University Hospital, Oslo, Norway; 14 Department of Nutrition, Harvard T.H. Chan School of Public Health, Boston, MA USA; 15 Clinical and Translational Epidemiology Unit and Division of Gastroenterology, Massachusetts General Hospital and Harvard Medical School, Boston, MA USA; 16 Department of Research, Telemark Hospital, Skien, Norway

**Keywords:** Colorectal carcinogenesis, Bowel cancer screening, Alcohol, Food frequency questionnaire, Gut microbiome, Causal mediation analysis

## Abstract

**Background:**

Alcohol consumption is one of the major risk factors of colorectal cancer (CRC), yet the mechanisms underlying this relationship, particularly the role of gut microbes, are not fully understood.

**Objective:**

To study associations of alcohol intake with the gut microbiome and colorectal lesions among CRC screening participants. Of particular interest was the potential role of gut microbes in mediating the association between alcohol intake and colorectal lesions.

**Methods:**

Screening participants with a positive faecal immunochemical test at ages 55–77 were eligible for the CRCbiome study. Alcohol intake was assessed using a validated, semi-quantitative food frequency questionnaire and linked with shotgun metagenome based gut microbial profiles to study associations with screen-detected colorectal lesions. The potential role of alcohol-associated gut microbes in mediating the association between alcohol intake and colorectal lesions was examined using causal mediation analysis.

**Results:**

Of 1468 participants with dietary data, 414 were diagnosed with advanced lesions. Alcohol intake was positively associated with advanced lesions in a dose-dependent manner (*p*_*trend*_ = 0.008), with odds ratio of 1.09 (95% confidence interval, 1.00, 1.19) per 10 g/day increase. Compared to non-consumers, those consuming alcohol were characterized by a distinct microbial profile, manifested as modest, but consistent, shifts in α- and β-diversity, and differentially abundant bacteria*.* A causal mediation analysis showed that 12% of the association between alcohol intake and advanced lesions was mediated by alcohol-associated gut bacteria.

**Conclusion:**

Alcohol consumption was associated with a distinct microbial profile, which partly explained the association between alcohol intake and advanced colorectal lesions.

*Trial registration*: The BCSN is registered at clinicaltrials.gov (National clinical trial (NCT) no. 01538550).

**Supplementary Information:**

The online version contains supplementary material available at 10.1007/s00394-026-03960-6.

## Introduction

Alcohol consumption increases the risk of cancer at multiple sites, particularly in the gastrointestinal system [1–3]. Recent global estimates suggest that about 1 in 20 cancers were attributed to alcohol consumption in 2020 [4]. With rising adult per capita consumption, especially in developing countries, these figures may increase [5]. The changing patterns of alcohol consumption among women represent a particular cause of concern [5].

Alcohol (ethanol) is efficiently absorbed in the upper gastrointestinal tract, mainly in the stomach and small intestine, before entering the liver via the portal vein [6]. The primary metabolic pathway involves alcohol dehydrogenase (ADH) and acetaldehyde dehydrogenase (ALDH), converting ethanol to acetaldehyde and acetate, respectively [6]. Alcohol can also be metabolized to acetaldehyde through the cytochrome P450 2E1 (CYP2E1) pathway [7]. Although the liver is the primary site for alcohol metabolism, some alcohol is also catabolized in the gastrointestinal tract, either by mucosal cells lining the gut or bacteria expressing enzymes involved in alcohol metabolism [7].

Alcohol may promote cancer directly or indirectly through its metabolites (acetaldehyde and acetate) and/or enzymes involved in its metabolism [7]. Their potential carcinogenic effects are multiple, encompassing genomic, biochemical, inflammatory and immune-modulatory mechanisms, among others [7]. Recently, the gut microbiome has emerged as a plausible pathway through which alcohol may promote cancer [8]. While it remains unclear whether and how alcohol contributes to carcinogenesis through this microbial pathway, plausible mechanisms demonstrated in model systems include the oxidation of residual ethanol to produce genotoxic acetaldehyde by some gut bacteria [9], or perturbation of the intestinal microbiome through a shift towards acetate consumption [6].

To explore the role of gut bacteria in alcohol-associated carcinogenesis, colorectal cancer (CRC) is particularly relevant. Not only are incidence rates of CRC highly connected to alcohol consumption (with approximately one in ten cases attributable to it [4]), but substantial data also support gut microbes as key players in the development [10] and early detection [11] of this disease.

In this study, we combined data on alcohol consumption with metagenome based taxonomic and functional profiles from participants in a large bowel cancer screening trial in Norway to shed light on this interplay. Our objectives included investigating the associations between alcohol consumption and screening-detected colorectal lesions, identifying microbial features linked to alcohol consumption in this population, and exploring whether the microbiome is a mediating factor in the alcohol-colorectal lesions relationship.

## Subjects and methods

### Bowel cancer screening in Norway (BCSN) and the CRCbiome study

The CRCbiome study is part of the Bowel Cancer Screening in Norway (BCSN) [12, 13], a pilot for the national screening program. BCSN is a randomized trial comparing once-only sigmoidoscopy with four rounds of biennial fecal immunochemical testing (FIT). Initiated in 2012, BCSN invited 139,291 participants, including 70,096 in the FIT arm. FIT-positive participants (≥ 15 µg hemoglobin/g feces) were referred for work-up colonoscopy.

Launched in 2017, the CRCbiome study invited 2700 participants during its four-year recruitment period, starting from the second FIT round. The long-term goal is to develop a microbiome-based biomarker to enhance FIT-based testing. FIT-positive participants were invited to join CRCbiome between receiving their FIT results and attending colonoscopy. Besides the invitation letter, participants received two questionnaires to be completed prior to colonoscopy: a food frequency questionnaire (FFQ) and a lifestyle and demographics questionnaire. Returning at least one questionnaire indicated consent, achieved by 1640 (61%) participants. Enrollment ages ranged from 55 to 77.

Both the BCSN and the CRCbiome study have been approved by the Regional Committee for Medical Research Ethics in South-East Norway (Approval no.: 2011/1272 and 63,148, respectively). The BCSN is also registered at clinicaltrials.gov (Clinical Trial (NCT) no.: 01538550).

### Study sample

**Fig. 1:**
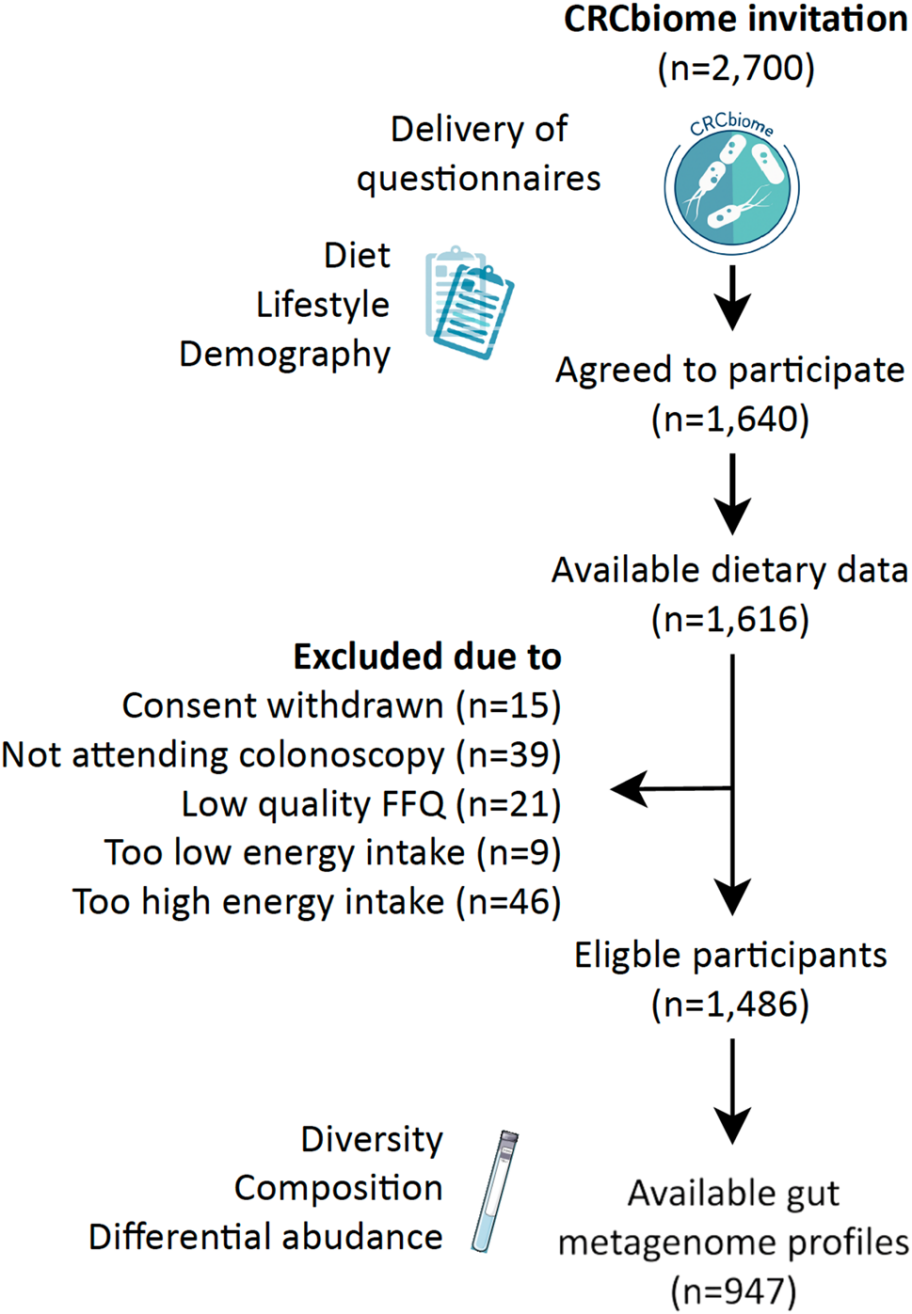
Flowchart of study participants. Abbreviations: FFQ; food frequency questionnaire

The current study included participants from the CRCbiome study with dietary information (n = 1616; see flowchart, Fig. 1). After excluding those who withdrew (n = 15), did not attend colonoscopy (n = 39), had a poor-quality FFQ (n = 21), or reported energy intake outside specified limits (low: < 600 kcal/day for women, < 800 kcal/day for men; high: > 3500 kcal/day for women, > 4200 kcal/day for men; n = 55 [14]), a total of 1,486 participants were eligible. Of these, 947 had gut metagenomic profiles from faecal samples; these had been preselected, prioritizing based on colonoscopy findings and including only those with sufficient sequencing data [11].

### Assessment of alcohol intake

Information on dietary intake, including alcohol, was obtained using a self-administered semiquantitative, 14-page FFQ, designed to capture the habitual diet during the preceding year. The questionnaire is developed by Department of Nutrition, University of Oslo [15–21], and has been validated for a variety of nutrients [15, 17, 20, 21] and food groups [17–21], including alcohol [15–17]. The questionnaire covers 256 foods and beverages, of which eight concern alcohol (plus one for non-alcoholic drinks). For each beverage type, participants were asked to record frequency of consumption and amount. Daily alcohol intake was calculated using the dietary calculation system KBS, developed at Department of Nutrition, University of Oslo, using the most recent database at the time, AE-18, which is an extended version of the official Norwegian Food Composition Table, version 2018 [22]. Alcohol intake was quantified both as ethanol (g/day and energy percentage (E%)) and by alcoholic beverage type (g/day). The alcoholic beverages were categorized as follows (with the standardized unit used in calculations given in brackets): ‘Wine’, including both red and white wine (150 mL); ‘beer’, comprising regular and light beer (330 mL); ‘spirits’, encompassing spirits, mulled wines like port and cherry, and liqueurs (40 mL); and ‘drinks’, which included cocktails, alcoholic cider, and alcohol-containing soft drinks (200 mL). The broader ‘drinks’ category was chosen due to the low overall consumption of these beverages. Additionally, the ‘non-alcoholic drinks’ category, consisting of non-alcoholic beers (330 mL), was evaluated for comparison purposes. One alcoholic unit was defined as 12 g of pure alcohol [23].

Prior to analyses, all questionnaires were reviewed and evaluated by trained personnel according to a standardized framework for quality control assessment developed by the study group [13].

### Outcome assessment

Outcome data were obtained from the BCSN database, containing detailed clinicopathological information on all colorectal lesions detected at work-up colonoscopy. The information was recorded by the responsible endoscopist using a structured reporting system. Based on the most severe finding at colonoscopy, participants were categorized into the following three diagnostic groups: advanced lesions, comprising CRC (any adenocarcinoma of the colon or rectum), advanced adenomas (any adenoma with villous histology, high-grade dysplasia or diameter ≥ 10 mm) and advanced serrated lesions (any serrated lesion with largest diameter ≥ 10 mm or with dysplasia); non-advanced adenomas; and controls, i.e., no CRC, adenoma nor advanced serrated lesions detected.

### Sample collection, library generation and shotgun metagenome sequencing

Protocols for sample collection, library generation and shotgun metagenome sequencing are detailed elsewhere [13]. In brief, DNA was extracted from 500 µl aliquots of left-over FIT sample buffer using the QIAsymphony DSP Virus/Pathogen Midikit (Qiagen, Hilden, Germany), employing a bead-beating lysis protocol. Purified DNA was eluted in 60 µl AVE buffer (Qiagen, Hilden, Germany). DNA concentration was measured on Qubit (Thermo Fisher Scientific, MA, USA), where samples with concentrations < 1.5 ng/µl were re-extracted from a second aliquot. FIT samples with concentrations ≥ 0.7 ng/µl were eligible for shotgun metagenomic library preparation, prioritizing the highest concentration from multiple aliquots.

Sequencing libraries were generated following the Nextera DNA Flex Library Prep Reference Guide, scaling reaction volumes to one-fourth of the reference. Pools of 240 samples were combined and size selected to achieve fragment sizes of 650–900 bp. Sequencing was performed on the Illumina NovaSeq system (Illumina Inc., CA, USA) using S4 flow cells, with each pool sequenced on a single lane, resulting in paired end 2 × 151 bp reads.

### Determination of taxonomic and functional profiles

Sequencing reads were processed to remove adapters and low-quality bases using trimmomatic (v0.36) [24] with the following trimming options: leading 20, trailing 20, minlength 50. Reads mapping to the human genome (hg38) and PhiX were removed using Bowtie2 (v2.3.5.1) [25]. Taxonomy and gene content were assessed using MetaPhlAn3 (v3.0.7) and HumanN3 (v3.0.0) [26], with the mpa v30 ChocoPhlAn 201,901 pangenome database, using the UniRef90 database to assign gene families to Metacyc pathways. Taxonomic abundance was evaluated at the species level. Pathway abundance was scaled by the number of quality-controlled reads per million.

### Assessment of covariates

Information on covariates was obtained using a self-administered, four-page questionnaire on lifestyle and demographic data, which has been described in detail previously [13]. Questions relevant to the current study concerned demographic factors (national affiliation, education, occupation and marital status), clinical factors (family history of CRC and diagnosis of chronic bowel disorders) and lifestyle factors (smoking and snus habits and physical activity level). Smokers and snusers were defined as self-reported regular or occasional users or those having quit consumption within the last ten years. Total amount of moderate to vigorous physical activity (minutes/week) was calculated by summing the time spent in moderate and vigorous activity, the latter weighted by a factor of two to best match recent guidelines [27–29]. Body mass index (BMI) was calculated based on self-reported weight (kg) and height (cm) obtained from the FFQ.

### Statistics

Descriptive statistics are presented as counts and percentages for categorical variables and as medians with percentiles for continuous variables, reflecting the non-normal distribution of many of these measures. Consistent with this, pairwise correlations between continuous measures were computed using Spearman’s correlation coefficients (*r*).

To study the association between alcohol intake and colorectal lesions, multinomial logistic regression analysis was conducted. Colonoscopy findings were categorized into advanced lesions, non-advanced adenomas and controls, according to the outcome definition given above. Alcohol (as ethanol in g/day) was categorized by consumption level (0 g/day, > 0–10 g/day, ≥ 10–20 g/day and ≥ 20 g/day) and by adherence to national guidelines (full adherence: 0 g/day, partial adherence: < 10 and 20 g/day for women and men, respectively, and non-adherence: ≥ 10 and 20 g/day for women and men, respectively [23, 27, 30]). Linear (per 10 g/increase/day) and exponential (per twofold increase) consumption was evaluated. For the alcohol subtypes, participants were categorized as consumers/non-consumers.

The selection of covariates was based on a priori knowledge on the relationship between alcohol intake and colorectal lesions [31–33], with all multinomial logistic regression analyses being adjusted for age, sex, national affiliation, screening center, education level, family history of CRC, smoking status, BMI and level of physical activity.

To study potential differential influence of alcohol intake on colorectal lesions by sex, separate analyses were conducted in women and men. Potential interactions were examined using the Wald test. Subgroup analyses were also conducted by precursor lesion subtype (advanced adenoma or advanced serrated lesion) and location (advanced proximal or advanced distal lesion).

As sensitivity analyses, the main association analyses were run with alcohol intake calculated as energy percentage (E%) rather than g/day, use of a multiple imputation approach for handling of missing data, additional adjustment for potential confounding factors (intake of red and processed meat, whole grains and dairy products) or the exclusion of participants without metagenome data (n = 539). The potential influence of leaving out participants with self-reported bowel disorders (n = 216) was also evaluated.

In line with the most recent statement from the American Statistical Association on p-values [34], emphasis was put on effect sizes, variation and uncertainty of the data rather than P-values in the interpretation of the results.

To identify microbial features linked to higher alcohol intake, associations with the following three measures were examined: α-diversity (Shannon and inverse Simpson indices), β-diversity (Bray–Curtis dissimilarity metric), and bacterial species and pathway abundance. Associations with α-diversity were examined using linear regression with log-transformed diversity indices as the dependent variable. β-diversity was evaluated by principal coordinate analysis (PCoA) and permutational multivariate analysis of variance (PERMANOVA) with 999 permutations, while effect sizes were calculated using partial omega-squared (Ω^2^) values. Differential abundance analyses were performed using microbiome multivariable associations with linear models (MaAsLin) 2 [35] with minimal prevalence of 0.1, normalization set to ‘none’ and ‘total sum scaling’ for bacteria and pathways, respectively and log2-transformation with pseudo-counts. Benjamini–Hochberg corrected p-values were applied. All analyses on microbial data were conducted in the study group as a whole and stratified by sex, using the same set of covariates as described above with the addition of sequencing depth. Other combinations of covariates were also evaluated (i.e. the addition of dietary risk or protective factors, presence of bowel disorders, as an indicator of bowel movement pattern, and antibiotic usage), but not included in subsequent analyses as they only marginally altered the results.

To evaluate the potential role of gut microbiome in mediating the association between alcohol intake and advanced lesions, a causal mediation analysis was applied. An alcohol-associated microbial score was developed as a summary measure of microbiome features linked to alcohol consumption. The score was constructed based on the output of adjusted differential abundance analyses, using the formula by Gevers, et al. [36], with zeros replaced by pseudo-counts to avoid computational issues. To minimize bias, a five-fold cross-validation approach was employed, calculating scores from non-overlapping subsets of the dataset.

In the causal mediation analysis, twofold increases in alcohol intake were treated as the independent variable, advanced lesions as the dependent variable and the alcohol-associated microbial score as the potential mediator. Non-advanced adenomas were grouped with colonoscopy-negatives for these analyses. The mediation analysis was adjusted for the same covariates as described previously, including the cross-validation partition identity. We used the R package mediation [37] as our main analytical approach, supplemented by medflex [38] as an alternative.

Statistical analyses were performed using R, version 4.1.0 (The R Foundation for Statistical Computing, Vienna, Austria), with the main packages reported in Supplementary Table 1. The significance level was set at 5%.

## Results

### Study population

Table 1 outlines the characteristics of the study population by alcohol intake. The median age of participants was 67 years, with a slight male predominance (56%). Compared to non-consumers (13% of participants), those consuming alcohol were more often male (48–76% vs. 34%) and affiliated with the screening centre in Bærum municipality (45–57% vs. 28%). Consumers of alcohol were characterized by a greater proportion of participants who were married or cohabiting (79–86% vs. 68%), employed (33–39% vs. 22%), and holding a university or college degree (41–52% vs. 29%). They also more often reported use of snus tobacco (5–12% vs. 3%) and higher levels of physical activity (135–180 vs. 45 min/week) than non-consumers.

**Table 1:** Key characteristics of the study population overall and by alcohol intake (n = 1486)^1^

Variables	Level of alcohol intake					
	n	Overall (n = 1486)	0 g/day (n = 187)	> 0–10 g/day (n = 592)	≥ 10–20 g/day (n = 361)	≥ 20 g/day (n = 346)
Age, years	1486	67 (62, 72)	67 (62, 73)	67 (62, 72)	66 (62, 72)	68 (62, 72)
Male sex, n (%)	1486	826 (55.6)	70 (37.4)	281 (47.5)	212 (58.7)	263 (76.0)
Screening centre, n (%)	1486					
Centre 1 (Moss)		774 (52.1)	135 (72.2)	323 (54.6)	166 (46.0)	150 (43.4)
Centre 2 (Bærum)		712 (47.9)	52 (27.8)	269 (45.4)	195 (54.0)	196 (56.6)
National affiliation, n (%)	1427					
Norwegian		1345 (94.3)	160 (89.4)	540 (94.7)	333 (96.0)	312 (94.3)
Non-Norwegian		82 (5.7)	19 (10.6)	30 (5.3)	14 (4.0)	19 (5.7)
Family history of CRC, n (%)	1347					
No		1092 (81.1)	133 (83.1)	442 (81.1)	272 (83.2)	245 (77.8)
Yes		255 (18.9)	27 (16.9)	103 (18.9)	55 (16.8)	70 (22.2)
Marital status, n (%)	1465					
Married/cohabiting		1171 (79.9)	123 (67.6)	460 (79.0)	308 (85.8)	280 (81.9)
Not married/non-cohabiting		294 (20.1)	59 (32.4)	122 (21.0)	51 (14.2)	62 (18.1)
Education, n (%)	1462					
Primary school		251 (17.2)	54 (29.8)	102 (17.5)	50 (14.0)	45 (13.2)
High school		580 (39.7)	75 (41.4)	243 (41.8)	144 (40.2)	118 (34.6)
University/college		631 (43.2)	52 (28.7)	237 (40.7)	164 (45.8)	178 (52.2)
Working status, n (%)	1464					
Employed		498 (34.0)	40 (22.1)	190 (32.6)	140 (39.1)	128 (37.4)
Retired/unemployed		966 (66.0)	141 (77.9)	393 (67.4)	218 (60.9)	214 (62.6)
Bowel disorder, n(%)	1451					
No bowel disease		1235 (85.1)	147 (82.6)	482 (84.0)	300 (83.8)	306 (89.7)
IBS		81 (5.6)	10 (5.6)	30 (5.2)	31 (8.7)	10 (2.9)
Celiac disease		18 (1.2)	3 (1.7)	8 (1.4)	4 (1.1)	3 (0.9)
IBD		24 (1.7)	5 (2.8)	11 (1.9)	7 (2.0)	1 (0.3)
Other		93 (6.4)	13 (7.3)	43 (7.5)	16 (4.5)	21 (6.2)
Smoking status^2^, n (%)	1462					
Non-smoker		1082 (74.0)	127 (70.2)	438 (75.3)	270 (75.4)	247 (72.4)
Smoker		380 (26.0)	54 (29.8)	144 (24.7)	88 (24.6)	94 (27.6)
Snus status^3^, n(%)	1405					
Non-snuser		1308 (93.1)	171 (97.2)	530 (95.3)	318 (92.4)	289 (87.8)
Snuser		97 (6.9)	5 (2.8)	26 (4.7)	26 (7.6)	40 (12.2)
BMI, kg/m^2^	1480	26 (24, 29)	27 (25, 30)	26 (24, 29)	26 (24, 29)	27 (24, 29)
Physical activity, min/week	1466	135 (0, 300)	45 (0, 195)	135 (0, 300)	180 (15, 390)	180 (45, 352)
Questionnaires completed prior to colonoscopy, n(%)	1486					
No		126 (8.5)	15 (8.0)	42 (7.1)	30 (8.3)	39 (11.3)
Yes		1360 (91.5)	172 (92.0)	550 (92.9)	331 (91.7)	307 (88.7)

BMI; Body mass index, CRC; colorectal cancer, g; gram, n; number

^1^Values are median (Q1, Q3) for continuous variables and n (%) for categorical variables

^2,3^To be defined as a smoker or snuser one had to be a regular or occasional user or having quit consumption within the last ten years

### Daily intake of alcohol

Daily intake of alcohol is presented in Table 2. The median intake of alcohol (as ethanol in g/day) was 9 g, with men at 13 g and women at 5 g. Adherence to national guidelines was relatively similar between sexes (68% in men, 65% in women). Wine was the most consumed beverage by women (74%), beer the most consumed by men (80%). The correlation between alcohol consumption and overall energy intake was weak (*r*_s_ = 0.22).

**Table 2:** Alcohol consumption in the study population as a whole (n = 1486) and by sex (660 women, 826 men)

	Percentiles				Adherent to guidelines^1^	Zero consumers	Correlation with energy intake
	25	50	75	100	n (%)	n (%)	*r*_s_^2^
*Overall*							
Alcohol, g/day	2.2	9.0	19	195	991 (67)	187 (13)	0.22**
Alcohol, E%	0.7	2.9	6.0	45	1003 (68)	187 (13)	-0.04
Alcohol units^3^/day	0.2	0.7	1.6	16	1083 (73)	187 (13)	0.22**
*Alcohol subtypes, g/day*							
Beer	0.0	33	140	3743	–	535 (36)	0.24**
Wine	0.0	36	108	837	–	417 (28)	0.06*
Spirits	0.0	0.0	1.2	223	–	1040 (70)	0.11**
Drinks, cider, etc	0.0	0.0	0.0	768	–	1240 (83)	0.10**
Non-alcoholic drinks, g/day	0.0	0.0	14	2000	–	1100 (74)	0.13**
*Men*							
Alcohol, g/day	3.8	13	25	195	563 (68)	70 (8)	0.17**
Alcohol, E%	1.2	3.6	7.2	45	503 (61)	70 (8)	-0.10*
Alcohol units^3^/day	0.3	1.1	2.1	16	610 (74)	70 (8)	0.17**
*Alcohol subtypes, g/day*							
Beer	16	80	203	3743	–	167 (20)	0.19**
Wine	0	35	108	837	–	247 (30)	0.04
Spirits	0	0	4,8	223	–	489 (59)	0.07
Drinks, cider, etc	0	0	0	768	–	667 (81)	0.07*
Non-alcoholic drinks, g/day	0	0	14	2000	–	591 (72)	0.11*
*Women*							
Alcohol, g/day	1.1	5.4	13	87	428 (65)	117 (18)	0.13*
Alcohol, E%	0.4	2.0	4.8	34	500 (76)	117 (18)	-0.12*
Alcohol units^3^/day	0.1	0.5	1.1	7.23	473 (72)	117 (18)	0.13*
*Alcohol subtypes, g/da*y							
Beer	0.0	0.0	45	1280	–	368 (56)	0.11*
Wine	0.0	41	108	837	–	170 (26)	0.09*
Spirits	0.0	0.0	0.0	78	–	549 (83)	0.04
Drinks, cider, etc	0.0	0.0	0.0	288	–	571 (87)	0.08
Non-alcoholic drinks, g/day	0	0	0	500	–	511 (77)	0.19**

E%; energy percentage, n; number, r_s_; Spearman’s correlation coefficient

^1^ < 10 g/day for women and < 20 g/day for men according to Nordic and national nutritional reccommendations [23, 27]; < 5 energy percentage (E%) for both sexes according to Nordic and national nutritional reccommendations [23, 27]; < 1 unit for women and < 2 units for men according to national food-based dietary guidelines [39]

^2^** < 0.001, * < 0.05

^3^1 unit set to 12 grams in line with national guidelines [23, 27]

### Alcohol intake and colorectal lesions

Compared to non-consumption, all levels of alcohol intake, and in particular high levels, were positively associated with advanced lesions (*p*_*trend*_ = 0.008, Table 3). The probability of advanced lesions increased by 9% per 10 g increase/day and 14% per twofold increase/day. Partial and non-adherence to guidelines were also positively associated with advanced lesions relative to not consuming alcohol, with odds ratios (ORs) of 1.91 (95% confidence intervals (CI) 1.22, 2.99) and 1.97 (1.23, 3.17), respectively. Notably, associations were stronger in women; for instance, women consuming ≥ 20 g/day had an OR of 4.81 (95% CI 2.18, 10.62) compared to 1.48 (0.74, 2.96) in men (*p*_*interaction*_ = 0.040). No associations between alcohol intake and non-advanced adenomas were detected.

**Table 3:** Odds ratios (ORs) and 95% confidence intervals (CIs) for presence of non-advanced adenoma^1^ and advanced lesions^2^ relative to controls by level of alcohol consumption in the study population as a whole (n = 1486) and by sex (660 women, 826 men)^3,4^

	Control (n = 548)	Non-advanced adenoma (n = 524)		Advanced lesions (n = 414)	
	n	n	OR (95% CI)	n	OR (95% CI)
*Overall*					
Level of intake					
0 g/day	88	65	Ref	34	Ref
> 0–10 g/day	226	202	1.16 (0.79, 1.70)	164	**1.81 (1.15, 2.85)**
≥ 10–20 g/day	126	131	1.31 (0.86, 2.00)	104	**1.99 (1.22, 3.27)**
≥ 20 g/day	108	126	1.30 (0.84, 2.02)	112	**2.19 (1.32, 3.63)**
*p*_*trend*_			0.20		**0.008**
Per 10 g increase/day			1.03 (0.94, 1.12)		**1.09 (1.00, 1.19)**
*Pcont*			0.52		**0.047**
Per twofold increase/day			1.03 (0.96, 1.11)		**1.14 (1.05, 1.23)**
*Pcont*			0.42		**0.002**
Adherence to guidelines					
Fully adhering (0 g/day)	88	65	Ref	34	Ref
Partially adhering (< 10/20 g/day)	291	275	1.16 (0.80, 1.70)	238	**1.91 (1.22, 2.99)**
Not adhering (≥ 10/20 g/day)	169	184	1.34 (0.89, 2.00)	142	**1.97 (1.23, 3.17)**
*Men*					
Level of intake					
0 g/day	31	22	Ref	17	Ref
> 0–10 g/day	87	111	1.58 (0.84, 2.97)	83	1.55 (0.78, 3.06)
≥ 10–20 g/day	65	73	1.37 (0.71, 2.67)	74	1.92 (0.95, 3.88)
≥ 20 g/day	84	99	1.32 (0.69, 2.51)	80	1.48 (0.74, 2.96)
*p*_*trend*_			0.99		0.48
Per 10 g increase/day			1.00 (0.91, 1.11)		1.04 (0.95, 1.15)
*Pcont*			0.92		0.37
Per twofold increase/day			0.99 (0.89, 1.09)		1.08 (0.98, 1.21)
*Pcont*			0.81		0.13
Adherence to guidelines					
Fully adhering (0 g/day)	31	22	Ref	17	Ref
Partially adhering (< 20 g/day)	152	184	1.49 (0.81, 2.74)	157	1.69 (0.88, 3.25)
Not adhering (≥ 20 g/day)	84	99	1.32 (0.69, 2.52)	80	1.47 (0.74, 2.93)
*Women*					
Level of intake					
0 g/day	57	43	Ref	17	Ref
> 0–10 g/day	139	91	0.96 (0.58, 1.58)	81	**2.07 (1.10, 3.90)**
≥ 10–20 g/day	61	58	1.46 (0.82, 2.57)	30	1.80 (0.87, 3.73)
≥ 20 g/day	24	27	1.65 (0.81, 3.35)	32	**4.81 (2.18, 10.62)**
*p*_*trend*_	57	43	0.05	17	**0.001**
Per 10 g increase/day			1.09 (0.91, 1.30)		**1.27 (1.06, 1.52)**
*Pcont*			0.36		**0.010**
Per twofold increase/day			1.09 (0.97, 1.22)		**1.22 (1.08, 1.39)**
*Pcont*			0.13		**0.002**
Adherence to guidelines					
Fully adhering (0 g/day)	57	43	Ref	17	Ref
Partially adhering (< 10 g/day)	139	91	0.96 (0.58, 1.58)	81	**2.09 (1.11, 3.93)**
Not adhering (≥ 10 g/day)	85	85	1.51 (0.89, 2.57)	62	**2.66 (1.37, 5.19)**

^1^Includes any adenoma (adenomatous polyp) not fulfilling the criteria of being advanced

^2^Includes advanced adenoma, defined as any adenoma with either villous histology (≥ 25% villous components), high-grade dysplasia or polyp size greater than or equal to 10 mm; advanced serrated lesions, defined as any serrated lesions with size ≥ 10 mm or dysplasia; and colorectal cancer, defined as presence of adenocarcinoma arising from the colon or rectum

^3^Odds ratios (ORs) and 95% confidence intervals (CIs) are obtained from multinomial logistic regression analyses adjusting for the following covariates: age (continuous), sex (except in the sex-specific analyses), national affiliation (Norwegian affiliation, non-Norwegian affiliation, missing), screening centre (centre 1, centre 2), education level (primary school, high school, college/university, missing), family history of CRC (yes, no, unknown/missing), smoking status (non-smoker, smoker, missing), BMI (continuous with missing set to median) and level of physical activity (continuous with missing set to median). Bold values indicate statistical significance at the level of 5%.

^4^Potential interactions of alcohol intake with sex was examined using the Wald test, resulting in the following p-values: ‘Level of intake’ (*p*-values in sequential order (2–4): 0.57, 0.82, 0.040), ‘Per 10 g increase/day’ (*p*-value = 0.10), ‘Per twofold increase/day’ (*p*-value = 0.24), ‘Adherence to guidelines’ (*p*-values for partial and non-adherence of 0.69 and 0.28, respectively)

Subgroup analyses by lesion subtype and location implied associations for both groups of precursor lesions, regardless of lesion location (Supplementary Table 2).

Sensitivity analyses with alcohol intake as energy percentage (E%), use of a multiple imputation approach for handling of missing values, inclusion of additional potential confounders and restricting the study population to those with metagenome data only (Supplementary Tables 3–6) produced similar results as in the main analyses. This was also the case for an analysis excluding participants with a self-reported bowel disorder (data not shown).

**Fig. 2:**
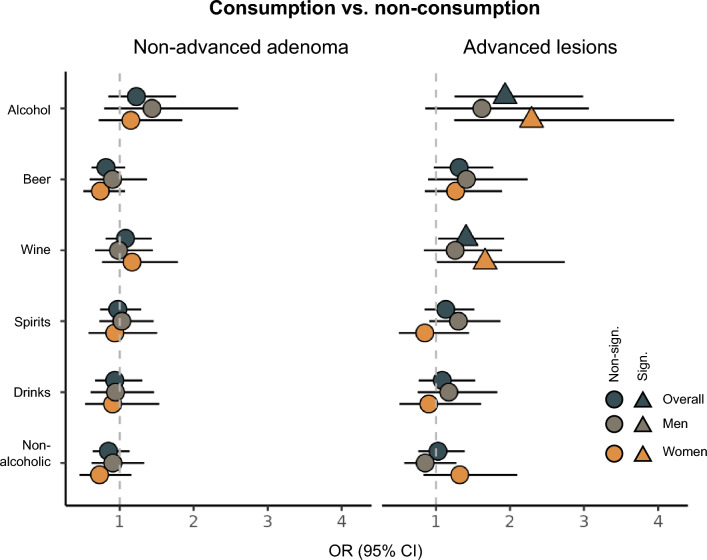
Odds ratios (ORs) and 95% confidence intervals (CIs) for presence of non-advanced adenoma and advanced lesions relative to controls for participants consuming vs. not consuming alcohol, as well as the different types of alcoholic beverages in the study overall (n = 1486) and by sex (660 women, 826 men). The non-consumers are used as reference category. Significant values are indicated by a triangle formation

In terms of beverage types, positive associations with advanced lesions were seen for consumers of wine (OR 1.41; 95%CI 1.03, 1.92) and beer (OR 1.31; 0.97, 1.77; Fig. 2), although the latter was not statistically significant. Stratifying the analyses by sex, the association for wine was stronger (and only statistically significant) in women, whereas the association for beer tended to be stronger for men, though it did not reach statistical significance. No associations with non-advanced adenomas were observed.

### Alcohol intake and gut microbial features

Metagenome data were available for 947 participants, revealing 787 microbial species (mean (SD) per sample: 88 (15.5)).

#### α-diversity

**Fig. 3:**
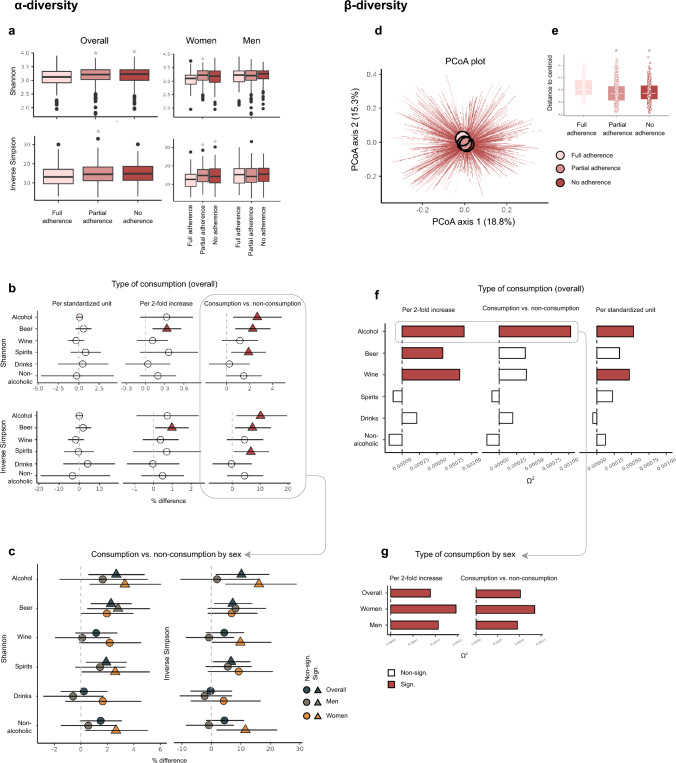
Associations of alcohol intake with the α-diversity indices Shannon and Inverse Simpson (**a**-**c**) and β-diversity based on the Bray–Curtis dissimilarity metric (**d**-**g**). **a** Levels of α-diversity by adherence to alcohol guidelines – overall (left) and stratified by sex (right). Significant differences relative to the reference group (“Full adherence”) are indicated by “*”. **b** Percentage (%) difference with 95% confidence intervals (CIs) in α-diversity per standardized unit increase (left), log2-fold increase (middle) and by consumption group (consumption vs. non-consumption) (right) for total alcohol intake and different types of alcoholic beverages. Significant differences are indicated by the combination of dark red colour and a triangle formation. **c** % difference with 95% CIs in α-diversity by consumption group (consumption vs. non-consumption) – overall (dark grey colour) and stratified by sex (with brown and yellow colour representing men and women, respectively). Significant differences are indicated by a triangular formation. **d** Principal coordinates analysis (PCoA) plot with the coloured circles representing group centroids of participants fully, partially and not adhering to alcohol guidelines. **e** Dispersion plot showing median distances to the group centroid for participants fully, partially and not adhering to alcohol guidelines. Significant differences relative to the reference group (“Full adherence”) are indicated by “*”. **f** Results of permutational multivariate analyses of variance (PERMANOVA) tests, visualized by means of omega-squared (Ω2) values, with total alcohol intake and different types of alcoholic beverages as input variables. Input variables were studied per twofold increase (left), by consumption group (consumption vs. non-consumption) (middle) and per standardized unit increase (right). Significant differences are indicated by dark red colour. **g** Results of PERMANOVA tests overall and by sex, visualized by means of Ω2 values, with total alcohol intake as input variable. The input variable was studied per twofold increase (left) and by consumption group (consumption vs. non-consumption) (right). Significant differences are indicated by dark red colour

In general, there was a weak, but statistically significant positive association between alcohol intake and both α-diversity indices (Fig. 3a-c and Supplementary Table 7). The shift in diversity became noticeable already at low intake levels. Compared to the non-consumers, those consuming alcohol had a 2.7 and 10.2% higher Shannon and Inverse Simpson index, respectively. Stratification by sex showed the associations between total alcohol intake and α-diversity to be statistically significant in women only. Still, looking at alcoholic beverage subtypes, statistically significant positive associations were observed for consumers of beer (overall and in men), wine (women), spirits (overall and in women) and non-alcoholic drinks (women).

#### β-diversity

Irrespective of the approach to quantifying alcohol intake, the microbial composition differed by intake level (PERMANOVA-derived *p*-values < 0.05, Supplementary Table 7. Consumers of alcohol displayed a small shift in microbial composition (Fig. 3d) and were less heterogeneous (Fig. 3e) than the non-consumers. Consuming alcohol was associated with microbial composition regardless of sex (Fig. 3g).

Consumption of wine, and to a lesser extent, beer, seemed to be related to microbial composition (Supplementary Table 7, Fig. 3f).

#### Differentially abundant bacteria and pathways

**Fig. 4:**
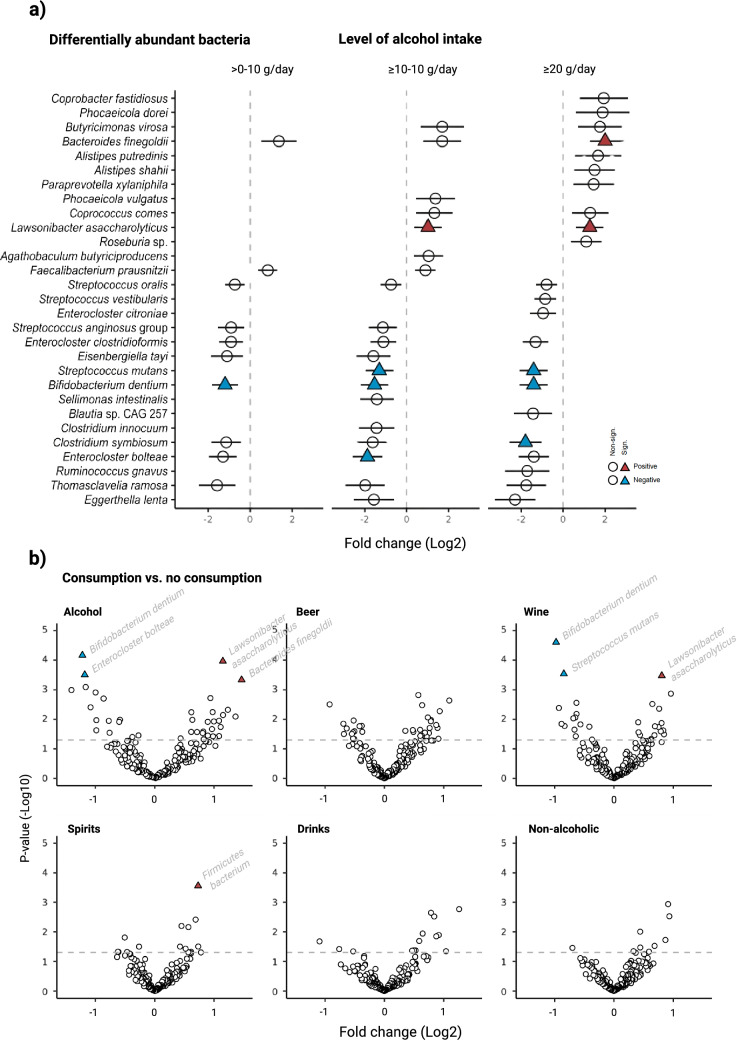
Differential abundance analyses of bacterial species by total alcohol intake and alcoholic beverage types. **a** Associations between alcohol intake and relative abundance assessed by microbiome multivariable associations with linear models (MaAsLin) 2. Values represent log2 fold changes with 95% confidence intervals. Species identified as significant predictors of alcohol intake after Benjamini-Hochberg (BH) correction in univariate testing are presented. Species remaining significant after multivariate adjustment are marked with a triangular formation, with colour indicating the direction of the association (red: positive, blue: negative). **b** Volcano plots showing multivariate adjusted associations for consumption of total alcohol and alcoholic beverage types with relative abundance assessed by MaAsLin 2. Values on the x-axis represent log2 fold changes, while values on the y-axis represent log10-transformed *p*-values. The dotted line is placed at -log10(0.05), indicating significance (non-corrected *p*-values). Species significant also after BH correction are marked with a triangular formation, with colour indicating the direction of the association (red: positive, blue: negative)

**Fig. 5:**
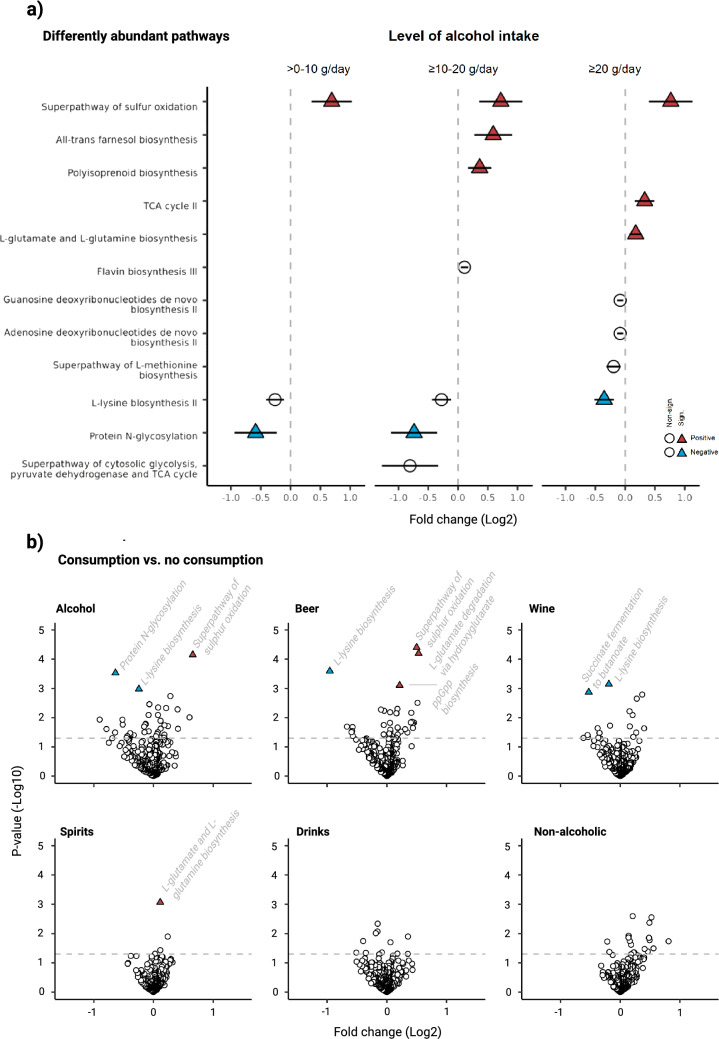
Differential abundance analyses of pathways by total alcohol intake and alcoholic beverage types. **a** Associations between alcohol intake and relative abundance assessed by microbiome multivariable associations with linear models (MaAsLin) 2. Values represent log2 fold changes with 95% confidence intervals. Pathways identified as significant predictors of alcohol intake after Benjamini-Hochberg (BH) correction in univariate testing are presented. Pathways remaining significant after multivariate adjustment are marked with a triangular formation, with colour indicating the direction of the association (red: positive, blue: negative). **b** Volcano plots showing multivariate adjusted associations for consumption of total alcohol and alcoholic beverage types with relative abundance assessed by MaAsLin 2. Values on the x-axis represent log2 fold changes, while values on the y-axis represent log10-transformed p-values. The dotted line is placed at -log10(0.05), indicating significance (non-corrected *p*-values). Pathways significant also after BH correction are marked with a triangular formation, with colour indicating the direction of the association (red: positive, blue: negative)

In total, 6 bacteria (2 positively and 4 negatively) and 7 pathways (5 positively and 2 negatively) were statistically significantly associated with at least one of the alcohol consumption categories (Figs. 4a, 5a). Of those predictive of the highest consumption category, 4 out of 5 bacteria (i.e. *L. asaccharolyticus*, *B. finegoldii*, *S. mutans* and *C. symbiosum*) and 3 out of 4 pathways (i.e. the tricarboxylic acid (TCA) cycle II, superpathway of sulphur oxidation and L-lysine biosynthesis II), remained statistically significant after mutual adjustment for the other bacteria and pathways, respectively (Supplementary Fig. 2). Several of the identified bacteria and pathways were inter-correlated (Supplementary Fig. 3). None of the identified bacteria or pathways showed signs of interaction with sex (Supplementary Fig. 4).

With regard to consumption of different alcoholic beverages (Figs. 4b, 5b), beer was statistically significantly associated with 4 pathways, wine with 3 bacteria and 2 pathways, and spirits with 1 bacterium. For the other beverage types, no statistical differences were detected.

### The gut microbiome as a potential mediator

**Fig. 6:**
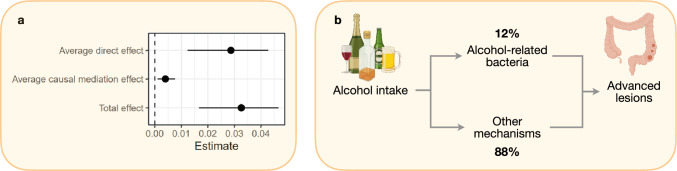
Causal mediation analysis. Alcohol intake (per twofold increase per day) was considered the primary exposure, the alcohol-associated gut microbial score the mediator, and advanced colorectal lesions as the outcome. **a** Point estimates and non-parametric bootstrap 95% confidence intervals, obtained using the percentile method with 1000 simulations, are reported for the total effect, average direct effect, and average causal mediation effect. **b** A direct acyclic graph showing the suggested relationship between alcohol intake, alcohol-associated gut microbes and advanced colorectal lesions with proportion mediated (%) for the average direct effect (ADE) and the average causal mediation effect (ACME). Analyses were conducted using the R package ‘mediation’ with the following adjustment set: age (continuous), sex, national affiliation (Norwegian affiliation, non-Norwegian affiliation, missing), screening centre (centre 1, centre 2), education level (primary school, high school, college/university, missing), family history of CRC (yes, no, unknown), smoking status (non-smoker, smoker, missing), BMI (continuous with missing set to median) and level of physical activity (continuous with missing set to median)

Based on output from differential abundance analysis, an alcohol-associated microbial score was developed to examine the potential mediating role of alcohol-related bacteria in colorectal carcinogenesis (see Supplementary Tables 8, 9 for bacterial species associated with alcohol intake overall and using the previously described five-fold cross-validation approach). The alcohol-associated microbial score partially explained the association between alcohol intake and advanced colorectal lesions (Fig. 6a), with a mediation proportion of 12.1% (3.1, 32.2; Fig. 6b), confirmed using an approach based on nested counterfactuals (here the proportion mediated was 12.2%, Supplementary Table 10). To assess potential differences in mediation proportion between women and men while maintaining power, we conducted a mediation analysis omitting sex as a covariate. This did, however, only marginally affected the results.

## Discussion

This study provides new evidence on the detrimental role of alcohol in colorectal lesion development. We found a positive association between alcohol consumption and advanced lesions detected at screening, even at modest intake levels. This association was particularly strong in women, while no clear association was observed in men. Alcohol consumers displayed a distinct gut microbial profile, which may partly explain the association with advanced lesions.

In this study, every 10 g increase in daily alcohol consumption was associated with a 9% increased probability of advanced lesions, consistent across lesion subtype and location. Our results align with the literature on precancerous colorectal lesions. Meta-analyses have demonstrated a 27% increased risk of adenoma per 25 g alcohol consumed per day [40], along with comparable increases in the risk of serrated polyps [41, 42]. Together, these results are in line with the literature on alcohol and CRC [31, 43, 44].

In our study, consumption levels even below 10 g/day were associated with advanced lesions. As such, our findings reinforce cancer prevention guidelines of complete abstinence to achieve the lowest possible risk [45–47].

The stronger association in women contrasts with prior literature. In the CUP 2016 meta-analysis from WCRF/AICR [31], as well as two pooled analyses (from UK and Japan, respectively) [48, 49], no particular heterogeneity was found between sexes. Women from Europe and Australia have the highest per-capita alcohol consumption worldwide, surpassing the global average by a factor of about two [5], with Norwegian women ranking among the top European countries for heavy episodic drinking in 2019 [50]. It is noteworthy that these regions also have the highest incidence rates of CRC among women, with Norway ranking at the top in 2020 [51]. The consumption pattern of participants included in the present study seems to mirror those of the general Norwegian population [52, 53] as well as the typical Norwegian socioeconomic gradient in the drinking pattern, high socioeconomic groups having the highest consumption [54, 55]. Whether country-specific trends in alcohol consumption pattern may account for divergence in findings remains a matter of speculation.

Our study demonstrated notable differences in the gut microbiome of participants consuming relative to not consuming alcohol, potentially of relevance to tumorigenesis. A link between alcohol intake and gut microbiome perturbations has been documented by others [56–60]. However, this has typically been studied in the context of chronic alcoholism [56, 57, 59] and/or presence of severe liver pathologies [57, 58] that could confound the alcohol-gut microbiome relationship. We observed that even modest alcohol consumption was associated with an increase in α- and a convergence of β-diversity. The diversity change has also been reported in studies of other Western populations [61–64], revealing alcohol as a strong source of gut microbiome variation.

We identified four bacterial species to be independently associated with alcohol consumption: *L. asaccharolyticus* and *B. finegoldii* were positively associated, whereas *C. symbiosum* and *S. mutans* were negatively associated. These results reproduce associations reported in the PREDICT study, which assessed microbial relationships with habitual diet in a large population, also finding *L. asaccharolyticus* and *C. symbiosum* to be strong determinants of alcohol consumption [64]. Among the identified bacteria, *C. symbiosum* has previously been suggested as a potential biomarker for CRC [65, 66], although the nature of this association remains unclear [11].

We found that the association between alcohol consumption and advanced lesions was partially mediated by the gut microbiome, accounting for a modest, yet conservative 12% of the total association. These findings are consistent with a potential role of alcohol-related microbial alterations in CRC development. However, it remains unclear how the relatively small concentrations of ethanol reaching the large intestine can induce microbial changes relevant to carcinogenic development. A conventional belief has been that the intestinal bacteria metabolized residual ethanol, leading to acetaldehyde accumulation and local damage [7, 8, 67]. However, this was recently questioned by Martino, et al*.* [6] suggesting that rather than metabolizing ethanol directly, gut bacteria responded to ethanol by activating acetate dissimilation. Our results also show a positive association between TCA cycle gene abundance and alcohol consumption. Acetate has recently received renewed attention in the context of CRC [7]. While traditionally seen as protective, recent evidence suggests that acetate may contribute to cancer cell growth by serving as a substrate for acetyl-CoA synthesis [7]. We also observed that alcohol consumption was associated with increased abundance of sulphur oxidation superpathway genes. While characteristic of a wide range of bacteria [68], it has been associated with CRC [68] and other gut disorders [69]. In combination, these results suggest that alcohol consumption may increase risk of CRC through alterations in bacterial acetate and sulphur metabolism.

A major strength of our study is the large microbiome sample set obtained through state-of-the-art methodology, coupled with validated exposure information. Access to clinically verified outcome data facilitated thorough investigations of relevant outcomes, minimizing misclassification bias. The study included only FIT-positive participants, resulting in a high lesion detection rate (63%); however, this selective inclusion may limit generalizability. High alcohol consumption was linked to high socioeconomic status in the study sample, potentially limiting the generalizability for populations in which alcohol is consumed differently across the socioeconomic classes. Additionally, the cross-sectional design restricts causal interpretations, making the results largely hypothesis-generating. While comprehensive lifestyle and demographic data facilitated detailed covariate adjustment, residual or reverse confounding cannot be ruled out. Also, participant selection based on colon bleeding may have introduced bias, potentially leading to uneven distribution of gastrointestinal morbidity across alcohol intake and outcome categories. This may be of particular concern for the present study, as some over-representation of former drinkers and “sick quitters” among the non-consumers, is to be expected [70]. However, excluding participants with self-reported gastrointestinal morbidity did not alter the observed associations.

To conclude, our study confirms the role of alcohol in the aetiology of CRC, identifying consistent associations between alcohol consumption and advanced lesions, particularly in women. Consuming alcohol was associated with a distinct gut microbial profile, which partly mediated the association between alcohol intake and advanced lesions. The potential role of alcohol-associated microbial alterations in cancer development should be further examined in prospective cohort studies with long-term follow-up. Such studies should investigate potential sex differences and ideally expand the repertoire of biological mechanisms by evaluating metabolic, inflammatory, and immune-modulatory pathways.

## Supplementary Information

Below is the link to the electronic supplementary material.

Supplementary file1.

## Data Availability

DNA sequencing data analysed in this study are deposited in the database Federated EGA under accession code EGAS50000000170 (https://ega-archive.org/studies/EGAS50000000170). Per participant consent, submitted FASTQ files exclude reads mapping to the human genome. The data are available under restricted access due to the sensitive nature of data derived from human subjects. Processing of data from this study must comply with the General Data Protection Regulation (GDPR). Access can be obtained by following the procedure described here: https://www.mn.uio.no/sbi/english/groups/roungegroup/crcbiome/. Requests for data access can also be directed to Trine B Rounge, trinro@uio.no.

## Abbreviations

ACME: Average causal mediation effect
ADE: Average direct effect
ADH: Alcohol dehydrogenase
AICR: American institute for cancer research
ALDH: Acetaldehyde dehydrogenase
BCSN: Bowel cancer screening in Norway
BMI: Body mass index
CRC: Colorectal cancer
CUP: Continuous update project
CYP2E1: Cytochrome P450 2E1 pathway
E%: Energy percentage
FFQ: Food frequency questionnaire
FIT: Faecal immunochemical test
KBS: Kostberegningssystem
MaAsLin: Microbiome multivariable associations with linear models
PCoA: Principal coordinate analysis
PERMANOVA: Permutational multivariate analysis of variance
Q: Quartile
TE: Total effect
WCRF: World cancer research fund
